# Comment on Boitrelle et al. The Sixth Edition of the WHO Manual for Human Semen Analysis: A Critical Review and SWOT Analysis. *Life* 2021, *11*, 1368

**DOI:** 10.3390/life12071044

**Published:** 2022-07-13

**Authors:** Francesco Pallotti, Francesco Lombardo, Donatella Paoli

**Affiliations:** Laboratory of Seminology—Sperm Bank “Loredana Gandini”, Department of Experimental Medicine, “Sapienza” University of Rome, 00161 Rome, Italy; francesco.pallotti@uniroma1.it (F.P.); francesco.lombardo@uniroma1.it (F.L.)

We wish to congratulate Boitrelle and colleagues for their comprehensive critical review on the Sixth Edition of the WHO Laboratory Manual for Human semen examination [1,2]. As we had also observed in a previous commentary [3], a critical drawback of the 6th edition of the manual is the return to the distinction between rapidly and slowly progressive motile spermatozoa. As Boitrelle and colleagues remarked, no recent data was added to highlight the clinical value of this distinction. On our part, we stressed the fact that that the categorization of the spermatozoa speed categories will become unavoidably an approximation. Therefore, the subsequent variability in semen analyses will ultimately reduce the standardization among centers, the exact opposite of the aim of the WHO Manual. It is also useful to remind that the other meaningful modification of the Sixth Edition is the strong rejection of the equation “5th percentile = Threshold”. It is well known that the overlap between fertile and infertile subjects increases as the total sperm number worsens [4,5]. Reference ranges and thresholds are useless if they are not inserted in the clinical context of the infertile couple. The interpretation of fertility based upon the fifth percentile alone is rather inadequate and should be finally proscribed. We do not agree that experienced clinical andrologists might encounter difficulties in accepting the absence of a clear threshold as in the work up of the infertile male semen results should be inserted in a clinical context, in the absence of which a treatment might not be beneficial [6].

In conclusion, we appreciated the work from Boitrelle and colleagues, as it presents a comprehensive discussion on the strengths and weaknesses of the WHO manual. This impacts directly on our daily practice as scientific attention must be drawn on every possible aspect capable of increasing the efficiency of the work up of the infertile couple.
